# Investigation of the palatine approach as a novel technique to block the canine maxillary nerve: a comparative cadaveric study

**DOI:** 10.1111/jsap.70115

**Published:** 2026-03-15

**Authors:** I. Antonopoulou, M. Fitzmaurice, P. Mannion, D. Bainbridge, C. Adami

**Affiliations:** ^1^ Department of Veterinary Medicine University of Cambridge Cambridge UK; ^2^ The Ralph Veterinary Referral Centre Marlow UK; ^3^ Cambridge Radiology Referrals Cambridge UK

## Abstract

**Objectives:**

To develop a palatine approach to the canine maxillary nerve and compare its feasibility and the spread of dye and contrast medium with that of the infraorbital technique.

**Materials and Methods:**

Ten canine cadaveric heads were used in this study. The maxillary nerve was approached bilaterally by inserting hypodermic needles into either the infraorbital or the palatine foramen on each side. Dye/contrast injections (1:1 solution) were followed by CT and anatomical dissections, performed to isolate the maxillary nerves and measure their stained portions. Procedural failure was defined as a nerve stain <6 mm in length. Descriptive statistics, analysis of proportions and comparison of either means or medians were used for data analysis.

**Results:**

The stained portion of the maxillary nerve was shorter for the palatine technique (0 (0 to 10) mm) than the infraorbital technique (35 (17 to 52) mm) (*P* = .015). Procedural failure rate was higher for the palatine technique (80%) compared to the infraorbital technique (20%; *P* = .025). For both techniques, imaging showed contrast spread along the nerve pathway in 70% of injections. Contrast medium contamination of nasal turbinates and nasal passages did not differ between approaches and was observed in 50% of palatine and 30% of infraorbital injections (*P* = .057).

**Clinical Significance:**

The novel palatine approach produced a higher procedural failure rate than the infraorbital technique and cannot be recommended as a suitable alternative to existing methods for canine maxillary nerve block.

## INTRODUCTION

Locoregional techniques are widely used in veterinary anaesthesia as part of multimodal pain management. The maxillary nerve block is an inexpensive technique used by both general practitioners and anaesthesiologists. Its effectiveness has been demonstrated for various procedures including teeth extractions, surgical treatment of brachycephalic obstructive airway syndrome, rhinoscopy and nasal biopsy (Beckman & Legendre, 2002; De Gennaro et al., 2022; Fizzano et al., 2017; Kim et al., 2021; Milella & Gurney, 2016).

The maxillary nerve originates as a branch of the trigeminal nerve. It exits the skull through the rostral alar foramen, runs across the wall of the pterygopalatine fossa ventral to the orbit and enters the maxillary foramen. It then exits the infraorbital canal as the infraorbital nerve, innervating the maxilla, teeth, nasal mucosa and muzzle. Smaller branches innervate the lower eyelid (zygomatic nerve), soft and hard palate (lesser and greater palatine nerves) and the mucosa of the ventral part of the nasal cavity and the maxillary sinus (caudal nasal nerve) (Couturier et al., 2005; König et al., 2014; Dyce, 2018).

The infraorbital approach to the maxillary nerve is intra‐oral, based on identification of the infraorbital foramen, palpated at the level of the third premolar and used as a landmark (Beckman & Legendre, 2002). A needle is inserted caudally through the buccal mucosa and advanced into the foramen to deposit the local anaesthetic in the infraorbital canal (Grubb & Lobprise, 2020). Replacement of the needle with an atraumatic catheter has been investigated as a potential substitute to prevent intraneural injection (Viscasillas et al., 2013).

Another intra‐oral approach performed with the mouth held open and various extra‐oral techniques, the most common of which is the sub‐zygomatic, has also been described (Beckman & Legendre, 2002; Grubb & Lobprise, 2020; Langton & Walker, 2017). Although none of these techniques has been established as a reference method, the infraorbital approach has been most extensively researched in dogs and has demonstrated clinical effectiveness for oral surgery (Cremer et al., 2013; De Gennaro et al., 2022; Fizzano et al., 2017; Viscasillas et al., 2013). Like the infraorbital nerve, the major palatine nerve is a branch of the maxillary nerve. Together with both the minor palatine and caudal nasal nerves, it originates from the pterygopalatine nerve arising from the ventral surface of the maxillary nerve, slightly rostral to the level of the pterygopalatine ganglion (Evans & de Lahunta, 2010; Evans & Kitchell, 1993). However, the major palatine foramen, through which the nerve exits the skull, is located closer to the main maxillary trunk than the infraorbital foramen, utilised for the infraorbital approach (Esteves et al., 2009). The effectiveness of a block is a function of the proximity of the local anaesthetic to the target nerve structure: the closer the injection site is to the main target, the better the chances for effectiveness at minimal drug doses and volumes (Read et al., 2024). An approach to the maxillary nerve using landmarks located near the main nerve trunk could therefore improve the quality of the block.

The aims of this study were to develop and describe a novel approach to the maxillary nerve using the palatine (P) foramen as a landmark, and to compare it with the infraorbital (IO) approach in terms of feasibility, the pattern of contrast medium distribution assessed by CT and the length of stained nerve segment after dye injection evaluated by anatomical dissection. It was hypothesised that using the palatine approach, the contrast/dye mixture would have a greater length spread and coverage of the maxillary nerve than the infraorbital approach.

## MATERIALS AND METHODS

A total of ten canine heads were obtained from medium‐to‐large size cadavers, which had been donated by dog owners to the Queen's Veterinary School Hospital to contribute to scientific research advancement. Ethical approval was granted by the Ethics and Animal Welfare Committee of the Department of Veterinary Medicine of the University of Cambridge (Ethical Approval Number: CR748). No information about the age, sex, weight, body condition score and clinical history of the dogs was available. The dogs had been humanely euthanised for reasons not related to the study, and their heads subsequently stored at −20°C, to be thawed 72 hours before the beginning of the study.

The study was conducted in two phases. Phase I (day 1) involved the determination of the anatomical landmarks for the novel approach, the injections of the dye/contrast mixture and the CT scans. Phase II (day 2) consisted of dissecting the heads to isolate the maxillary nerves and measure both their total length and the length of their stained portions.

### Phase I

A preliminary anatomical study was conducted on three medium‐to‐large‐sized canine skulls. Hypodermic needles were inserted through the foramina along both the infraorbital and palatine canals until they exited at the caudal end. Thereafter, the portion of each needle spanning from the insertion site to its exit was stained with a permanent marker pen and measured with a ruler to determine the required insertion length, and therefore the most appropriate needle selection, for both approaches.

The skulls were also used to determine the injectate volume based on the size of each head, since the cadavers were unavailable for body weight measurement. In skulls of dogs estimated to weigh approximately 25 kg in life, the measured distance between the median palatine raphe and the first molar was approximately 25 mm (Beeston et al., 2022). Using this distance as a surrogate for body size, the total volume injected at each site was calculated to correspond to 0.05 mL/mm of measured distance, aiming at a minimal volume of 0.05 mL/kg for performing maxillary nerve block in a living dog (Beckman & Legendre, 2002; Langton & Walker, 2017; Viscasillas et al., 2013).

The maxillary nerves were approached using one of two intra‐oral techniques, the P and the IO.

**FIG 1:**
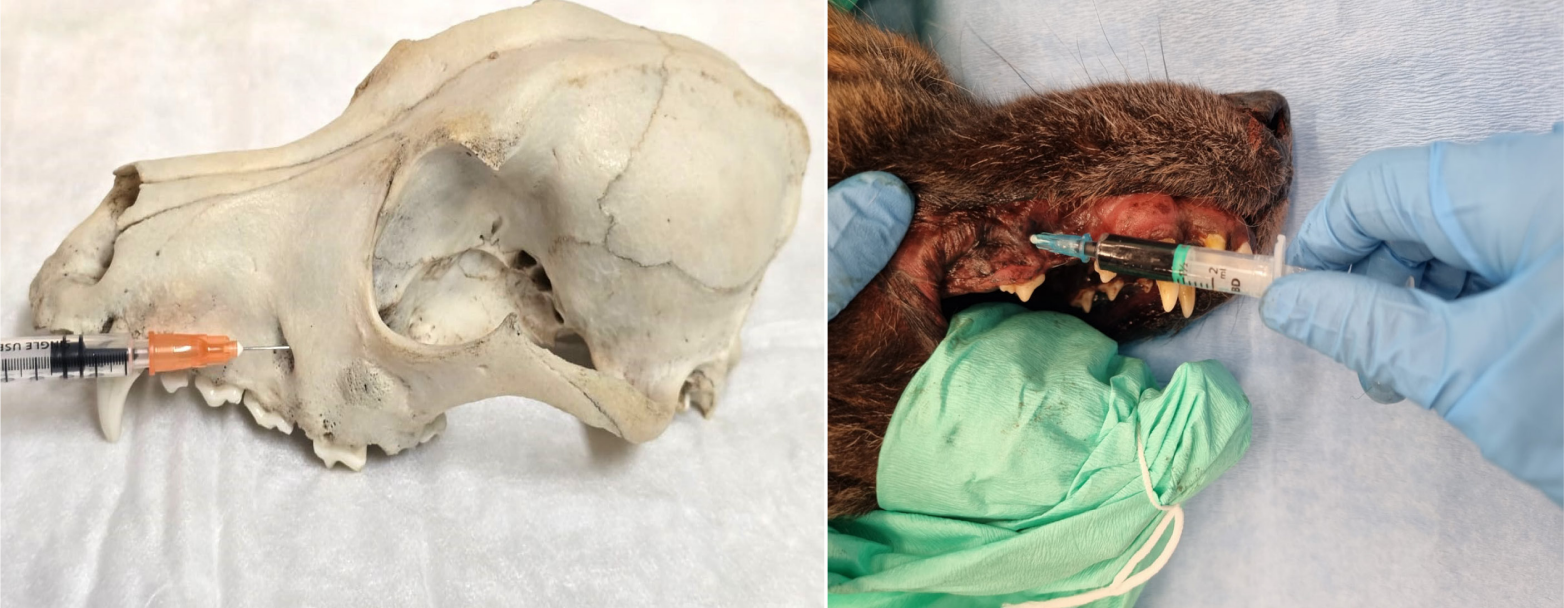
The infraorbital approach. The infraorbital approach to the maxillary nerve, demonstrated on a skull (left) and in a cadaver used in the study (right).

For the IO‐technique, the heads were placed in lateral recumbency. The needles were inserted intra‐orally into the infraorbital foramen, located at the level of the third premolar, parallel to the long axis of the skull and advanced for 15 mm (Fig. 1).

**FIG 2:**
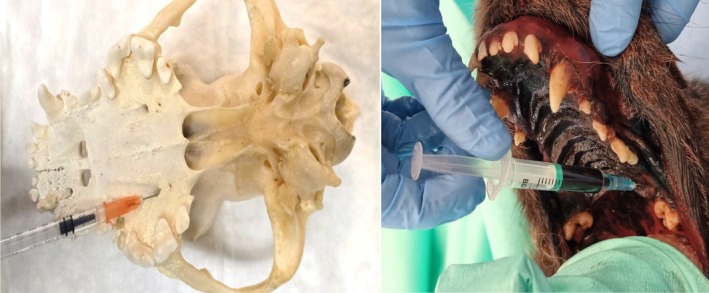
The palatine approach. The palatine approach to the maxillary nerve, demonstrated on a skull (left) and in a cadaver used in the study (right).

For the P‐technique, the heads were placed in dorsal recumbency with the mouth held open by an assistant. The needles were inserted parallel to the long axis of the skull, into the palatine foramen, located at the level of the first upper molar tooth and midway between the tooth and the median palatine raphe. The needle was advanced 15 mm, maintaining a direction parallel to the median palatine raphe (Fig. 2). In the first two heads, after needle insertion and prior to volume injection, the mucosa of the hard palate was incised to check the proper positioning of the needle body within the palatine canal.

Each head was injected using both techniques: one on the left side and the other on the right. Side allocation was determined using a randomisation app (Chooser, Bon app & T LTD), set in blocks of two to ensure that both techniques were always used within the same head. The injections were performed with hypodermic needles (Microlance, 23‐Gauge, 1.5 cm, country) and a 1:1 mixture of methylene blue (Methylene Blue 1% aqueous solution, Atom Scientific, UK) and the radio‐opaque contrast medium Ioxehol (Omnipaque 350; GE Healthcare, Germany).

A single investigator (IA) was responsible for the identification of the anatomical landmarks and the injection of the dye mixture. Another investigator (CA) was responsible for measuring and recording the distance between the median palatine raphe and the first molar tooth, and of subjectively confirming the correct needle placement for each approach. A third investigator (MF) was responsible for managing the randomisation app, calculating the total injection volume, preparing the mixture in a syringe and documenting findings.

Data recorded included the dog breeds, the distance between the median palatine raphe and the first molar, the injection volumes, whether difficulties were subjectively encountered while locating the anatomical landmarks (yes or no), and subjectively perceived resistance to injection (yes or no). Following completion of injections, a CT scan was performed on each head, positioned in ventral recumbency.

All scans were obtained with a Toshiba Aquilion 16‐slice CT machine and evaluated by the same veterinary diagnostic imaging board‐certified specialist (PM). Upon completion, the heads were moved to an appropriate, temperature‐controlled area (+4°C) in the *post mortem* room, awaiting anatomical dissection the next day.

### Phase II

The anatomical dissections took place in the *post mortem* room of the department. All the dissections were performed by two investigators, (DB) and (CA). Two investigators (IA and CA) measured the total length of the maxillary nerves, the length of the nerve‐stained portions and the radius of dye spread from the targeted nerve to the surrounding tissues. All measurements were performed with a ruler in mm. One investigator (MF) recorded the data.

For the dissections, the zygomatic arch was identified, and an incision was made on the skin at the ventral aspect of the zygomatic arch with a scalpel blade attached to a scalpel. The surrounding soft tissues, including subcutaneous layers, muscles and ligaments, were excised. A part of the zygomatic arch and then a segment of the coronoid process were removed to reveal the route of the maxillary nerve, arising from the rostral alar foramen to the rostral rim of the infraorbital foramen.

Once the maxillary nerve was exposed, its whole length was measured from the rostral alar foramen to the rostral aspect of the infraorbital foramen. Thereafter, the length of the stained nerve was measured in mm, by placing the ruler first on the rostral rim of the infraorbital foramen and directing the ruler caudally. Any dye and/or contrast contamination of the anatomical structures surrounding the target nerve (vascular structures, lymph nodes, salivary glands, parotid gland/area, nasal turbinates and nasal passages and subcutaneous or sub‐mucosal layers adjacent to the needle entry point) was recorded as a binary outcome variable (yes or no).

### Statistical analysis

During data analysis, the procedural failure rate was defined as stain extending less than 6 mm along the maxillary nerve (Stathopoulou et al., 2018).

The premise on which the sample size was calculated was that the P approach was expected to result in longer nerve staining than the IO approach. In order to detect with either the Mann–Whitney test or the *t*‐test a difference in length of staining between the two approaches, with estimated means of 6 mm for IO and of 9 mm for P, ± standard deviation (SD) of 2.4 mm (equal to 40% of 6 mm), α value: .05, β value: .2 and power: .8, the minimal number of injections per technique was identified as 10. Since each head was injected bilaterally, the minimal number of heads was identified as 10.

The Shapiro–Wilk test was used to assess normality and analyse the distribution of continuous data. Descriptive statistics, analysis of proportions and comparison of either means or medians were used for data analysis. Either the Fisher exact test or the chi‐square test, depending on whether two‐way contingency tables applied to the specific set of data, was used to identify associations between treatment group (either P or IO) and categorical variables. Either the Mann–Whitney test or the paired *t*‐test, depending on data distribution, was used to compare treatment groups with respect to continuous variables. Commercially available software was used (SigmaStat version 4.0 and SigmaPlot version 10, Systat Software UK Ltd; and SPSS version 28, IBM, Armonk, New York, USA). Statistical significance was set at *P* values < .05.

## RESULTS

Continuous data are presented as either means ± SD or medians and interquartile ranges, depending on data distribution. The represented dog breeds were German shepherd (*n* = 3), Staffordshire bull terrier (*n* = 3), golden retriever (*n* = 1), bull mastiff crossbreed (*n* = 1), cocker spaniel (*n* = 1) and border terrier (*n* = 1).

The distance between the median palatine raphe and the first molar and the volume of injectate were 24.8 ± 5.1 mm and 1.24 ± 0.25 mL, respectively (Table 1). Based on the preliminary anatomical study conducted on canine skulls, a needle insertion length of 15 mm appeared sufficient for the largest available skull to reach the full length of both the infraorbital and palatine canals. The IO approach was performed on the left side in 7/10 heads (70%), and on the right side in the remaining 3/10 (30%). No difficulties were subjectively reported for either technique with respect to the identification of the anatomical landmarks. Resistance during injection was subjectively perceived after 6/10 (60%) P‐technique injections and 0/10 (0%) after IO‐technique injections.

**Table 1:** Details of contrast medium/dye injections performed to approach the maxillary nerve using two different techniques (the palatine and infraorbital) in 10 canine cadaver heads

	Dog 1		Dog 2		Dog 3		Dog 4		Dog 5		Dog 6		Dog 7		Dog 8		Dog 9		Dog 10	
Median raphe – first molar distance (mm)	30		20		25		28		25		32		22		30		16		20	
Maxillary nerve length in mm	70		55		80		100		78		70		60		70		60		55	
Total injection volume per site (mL)	1.5 mL		1 mL		1.25 mL		1.4 mL		1.25 mL		1.6 mL		1.1 mL		1.5 mL		0.8 mL		1 mL	
Approach	IO	P*	IO	P*	IO	P	IO	P	IO	P	IO	P	IO	P	IO	P	IO	P	IO	P
Difficulty subjectively encountered locating anatomical landmark	N	N	N	N	N	N	N	N	N	N	N	N	N	N	N	N	N	N	N	N
Ease of needle placement	Eas	Une	Eas	Une	Eas	Eas	Eas	Une	Eas	Eas	Eas	Eas	Eas	Eas	Eas	Une	Eas	Eas	Eas	Eas
Subjectively perceived resistance to injection	N	N	N	N	N	Y	N	Y	N	Y	N	Y	N	Y	N	Y	N	N	N	N
Contrast distribution	Mx	Cd	Mx	Mx	Cr	Mx	Cr	Cd	Cr	Mx	Mx	Mx	Mx	Dif	Mx	Mx	Cd	Mx	Mx	Mx
Dye distribution	Unst	Cd	Cr	Unst	Cr	Unst	Unst	Mx	Mx	Unst	Cr	Unst	Mx	Unst	Cr	Unst	Mx	Unst	Mx	Unst
Nerve length stained (dye) in mm	0	67	35	0	45	0	0	40	55	0	40	0	23	0	70	0	15	0	35	0

Mixed pattern: cranial and caudal spread; Diffuse: along the path; Linear: along the nerve; Caudal: caudal to the site of injection; Cranial: cranial to the site of injection

Details of contrast medium/dye injections performed to approach the maxillary nerve using two different techniques (the palatine and infraorbital) in ten canine cadaver heads

* In these two heads, after needle insertion and prior to injection, the mucosa of the hard palate was incised to check the proper positioning of the needle body within the palatine canal

### Anatomical dissections

The portion of the stained maxillary nerve was shorter for the P‐technique [0 (0 to 10) mm] than for the IO‐technique (35 (17 to 52) mm) (*P* = .015). As a result, the procedural failure rate was greater for the P‐technique than for the IO‐technique (8/10, 80% and 2/10, 20%, respectively; *P* = .025).

Contamination of the salivary glands with dye did not differ between treatment groups and was detected after 3/10 (30%) P‐technique injections and 2/10 (20%) IO‐technique injections (*P* = 1). A greater proportion of IO‐technique (9/10, 90%) than P‐technique (2/10, 20%) injections resulted in contamination of the subcutaneous layers adjacent to the needle entry point (*P* = .005). Other anatomical structures contaminated with methylene blue after P‐technique injections were the sub‐palatine soft tissues (1/10, 10%), the regional (palatine) lymph nodes (1/10, 10%) and the palatine and facial arteries (1/10, 10%).

None of the six P‐technique injections during which resistance was perceived resulted in staining of any length of the maxillary nerve.

### Evaluation by CT imaging

**FIG 3:**
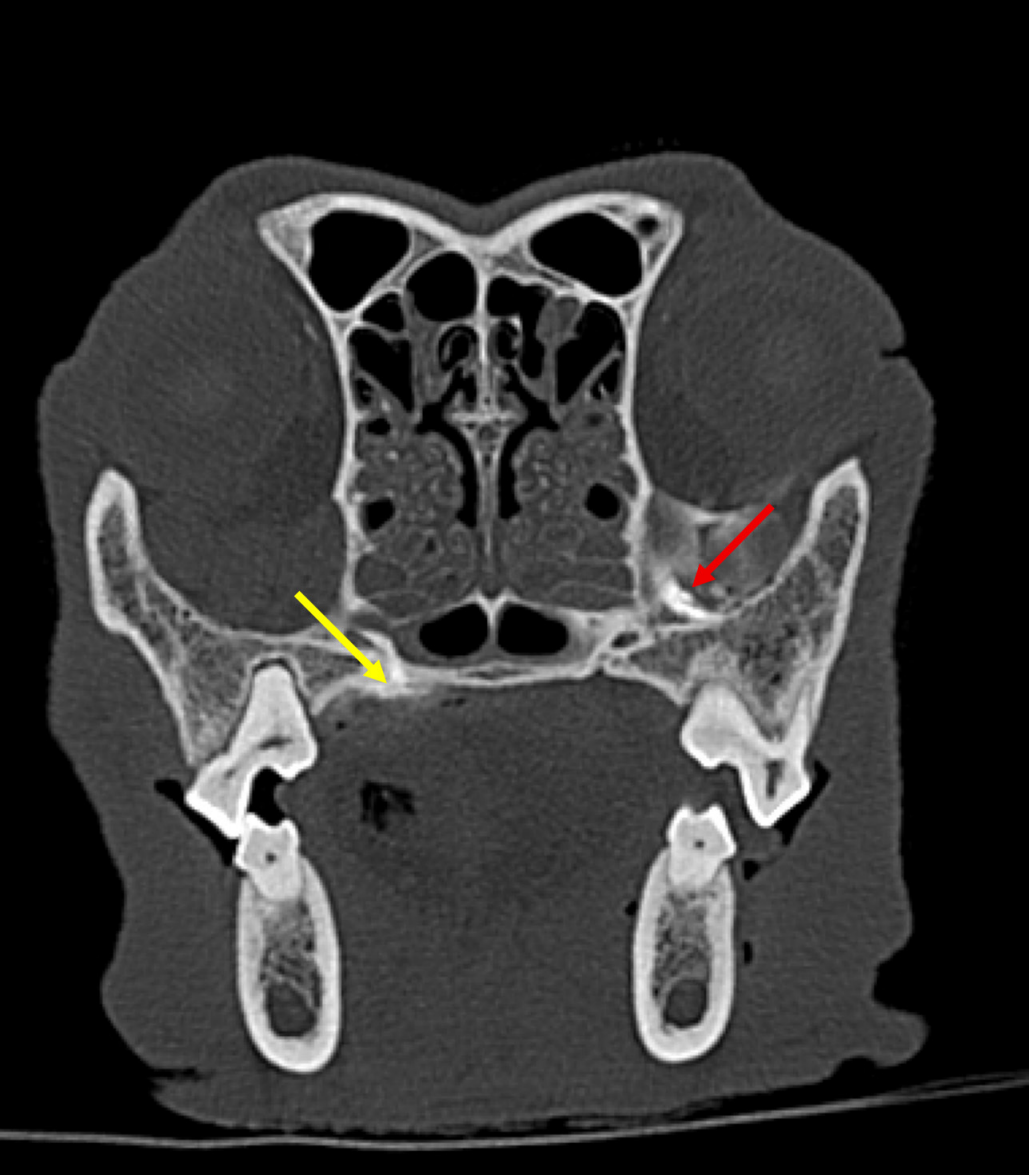
Successful palatine and infraorbital blocks. Yellow arrow: Successful deposition of dye/contrast along the maxillary nerve following a successful palatine approach. Red arrow: Successful deposition of dye/contrast along the maxillary nerve following a successful infraorbital approach.

Two types of contrast spread patterns were identified along the maxillary nerve path: linear and diffuse. Imaging revealed the presence of contrast spread along the nerve path following 7/10 (70%) of both IO‐technique and P‐technique injections (Fig. 3), with a pattern that was, for both techniques, linear in 6/7 (86%) cases and diffuse in the remaining 1/7 (14%). The distribution of contrast medium with respect to the needle entry point was cranial in 0/10 (0%) of the P‐technique and in 3/10 (30%) of the IO‐technique injections, caudal in 2/10 (20%) of the P‐technique and 1/10 (10%) of the IO‐technique injections, and mixed (both cranial and caudal) following 8/10 (80%) P‐technique and 6/10 (60%) IO‐technique injections, with no significant differences between approaches (*P* = .232).

**FIG 4:**
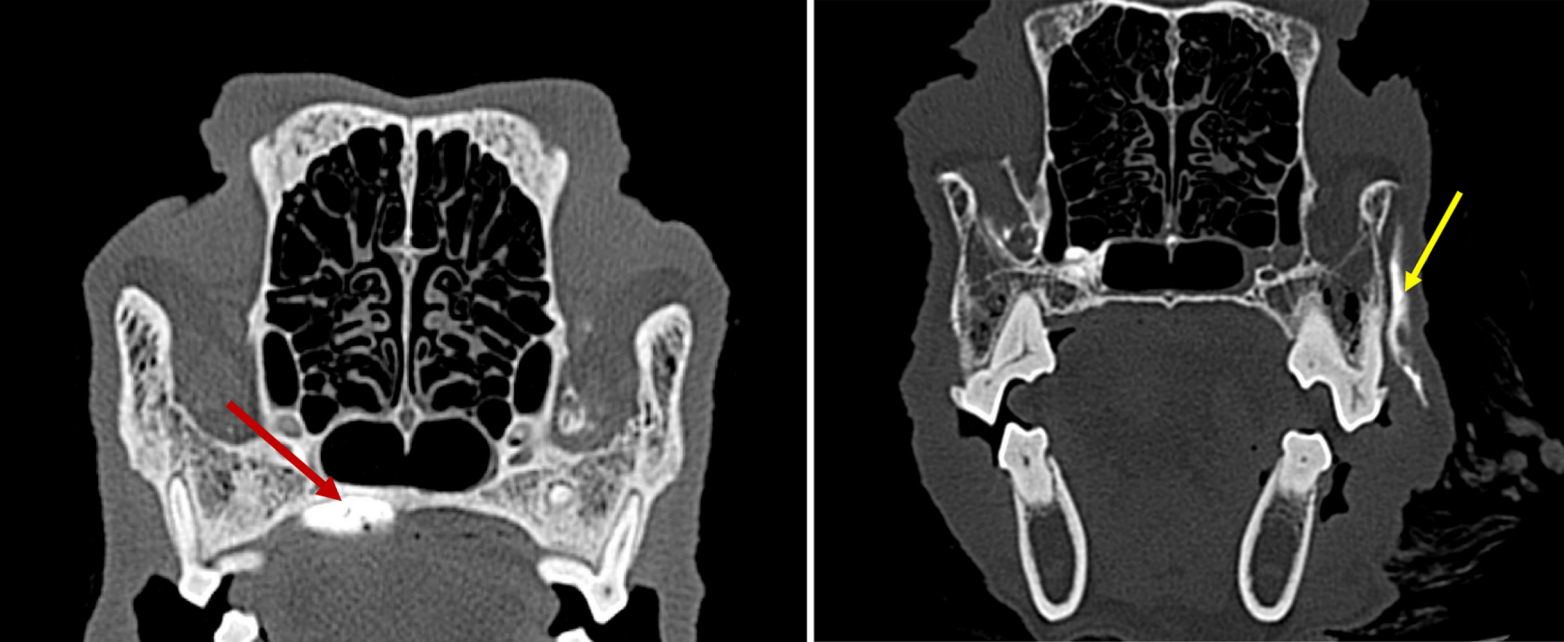
Contamination of tissues after unsuccessful palatine and infraorbital blocks. Red arrow: Contamination of the mucosa after a non‐successful palatine approach. Yellow arrow: Contamination of the surrounding soft tissues after a non‐successful infraorbital approach.

Contamination of nasal turbinates and nasal passages with contrast medium was observed in 3/10 (30%) of IO‐technique and 5/10 (50%) of P‐technique injections, with no differences between the two techniques (*P* = .057). The area around the parotid salivary gland showed contrast medium contamination following 2/10 (20%) P‐technique and 1/10 (10%) IO‐technique injections, with no differences between approaches (*P* = 1). The proportion of injections resulting in contamination of the soft palate sub‐mucosal area was greater for the P‐technique (80%, *n* = 8) than for the IO‐technique (10%, *n* = 1) approach (*P* = .005) (Fig. 4). Of the eight injections in the P‐technique group that resulted in contamination of the soft palate sub‐mucosal area, high resistance to injection was subjectively perceived in five (62.5%).

## DISCUSSION

Our findings show that approaching the maxillary nerve through the palatine foramen (P‐technique) is not a suitable alternative to the well‐established infraorbital approach (IO‐technique), as demonstrated by the greater procedural failure rate of the novel technique compared to the reference one.

The greater procedural failure observed for the P‐technique compared to the IO‐technique may be explained by the anatomical differences between the two anatomical landmarks. Although difficulties in identifying the palatine foramen were not specifically reported, the infraorbital foramen is the most prominent and easily palpable structure on the lateral aspect of the maxilla, while the palatine foramen is less accessible and anatomically smaller in diameter (Dyce, 2018). This could have contributed to the increased resistance encountered during half of the injections with the palatine approach.

Perceived resistance and injection pressures as high as 25 Pounds per square inch (PSI) while performing nerve blocks have been associated with intraneural injection of local anaesthetics, a complication that could lead to severe neural injury and result in persistent or transient neurological deficits (Hadzic et al., 2004). Injection pressures were not measured in the current study; however, neither the anatomical dissections nor the CT suggested that any accidental intraneural injections had occurred. Moreover, injection pressure is likely to differ between cadavers and living animals. Another reason for increased resistance while performing oral nerve blocks is the presence of calcifications in the injection areas, which in geriatric humans are common in the maxillary sinus and peri‐mandibular tissues (Syed, 2024). Nevertheless, it is worth mentioning that the CT scans of the heads failed to detect calcifications in the peri‐palatine area.

A common finding for both approaches was the contamination of the surrounding tissues with both dye and contrast medium. Studies conducted in humans showed that the distribution of both methylene blue and local anaesthetics is characterised by lesser spread in living patients compared to cadavers, potentially due to the uptake by lymphatic and blood circulation, as well as *post mortem* autolysis (Kull et al., 1997; Mowbray & Wong, 1988). In addition, the spread of both dye and contrast medium may be affected by the preservation status of the cadavers. It is therefore possible to speculate that peri‐neural tissue contamination may not occur, or may be limited, in living dogs following both infraorbital and palatine injections.

In the absence of data pertaining to the dogs' body weight, the volume injected was determined based on the size of their heads. Although there are no clear guidelines on effective injection volumes for blocking the maxillary nerve, the literature indicates that volumes as high as 0.05 mL/kg would be effective for this purpose (Beckman & Legendre, 2002; Langton & Walker, 2017; Viscasillas et al., 2013). One study suggested 0.3 to 0.6 mL of bupivacaine 0.5% as a volume range sufficient to produce a successful block for oral procedures in dogs weighing 6 to 25 kg (Beckman & Legendre, 2002). Other authors reported the use of volumes of local anaesthetics ranging from 0.5 to 1 mL per site of injection to block the maxillary nerve in medium‐sized cadavers weighing approximately 10 to 20 kg (Langton & Walker, 2017; Viscasillas et al., 2013). Based on their breeds, the cadavers used in the current study were estimated to weigh between 10 and 30 kg; given that the total injection volume used was 1.24 ± 0.25 mL, this volume is comparable to those reported in the literature.

Consistent with previous studies, success in the current study was defined as a minimum length of 6 mm for the stained portion of the maxillary nerve (Stathopoulou et al., 2018). The literature suggests that, in order to prevent impulse transmission, the local anaesthetic should spread for at least three consecutive nodes of Ranvier, which corresponds to approximately 6 mm in large mammalian nerve fibres (Raymond et al., 1989). However, other authors suggested that the distribution of dye in cadavers may not accurately reflect the spread of local anaesthetic in living animals and concluded that nerve exposure for a length shorter than 6 mm may be sufficient to achieve analgesia in a clinical setting (Bardell et al., 2010; Stathopoulou et al., 2018).

Although nerve staining clearly identified the infraorbital approach as the most effective technique, the proportion of injections resulting in contrast spread along the nerve pathway, as demonstrated by imaging, was the same for both approaches. While it is difficult to offer a definitive explanation for this finding, the potential instability of the dye/contrast mixture following injection, leading to variability in the distribution of the dye and contrast medium within and along the tissues, cannot be entirely ruled out.

A potential limitation of this study is the level of expertise of the investigator who performed all the injections. One study demonstrated that the variability in identifying the correct point of needle insertion for posterior lumbar plexus and sciatic nerve blocks was greater in inexperienced practitioners in comparison to experienced ones (Grant et al., 2003). In the current study, identification of the anatomical landmarks and perineural injections were performed by a specialist‐in‐training with an intermediate degree of experience. It is possible that a more experienced anaesthetist could have produced a different outcome; nevertheless, considering that dental nerve blocks are often performed in general practice, feasibility and ease of locating the anatomical landmarks are desirable features for maxillary block techniques.

In addition to technical pitfalls, insufficient nerve staining may have resulted from inadequate needle depth. Although 15 mm appeared appropriate based on preliminary anatomical studies on canine skulls, the presence of soft tissues in the cadaver heads, as well as potentially larger dog breeds included, may have contributed to increased variability, necessitating longer insertion lengths.

Another probable limitation could be identified as the 24‐hour delay between the injections and the cadaveric dissections, potentially contributing to further spread of the solution and post‐injection contamination of the perineural areas. This delay was dictated by both the time required to scan all the heads and the unavailability of the *post mortem* room outside regular working hours.

Lastly, CT‐guided needle insertion might have improved the accuracy of needle position prior to injection. However, because oral blocks are commonly performed with a blind technique, an approach requiring diagnostic imaging confirmation would not have been applicable to clinical practice.

In conclusion, based on the higher procedural failure rate, the palatine approach to the canine axillary nerve cannot be recommended as an alternative to the infraorbital approach.

Further studies are required to develop more successful approaches to the canine maxillary nerve, with a view to their use in clinical practice.

## Author contributions

I. Antonopoulou: Conception and design of the study, data collection and manuscript draft writing. M. Fitzmaurice: data collection and record, manuscript revision. P. Mannion: Intellectual contribution to study design, CT scans interpretation and manuscript revision. D. Bainbridge: Intellectual contribution to study design, anatomical dissections and manuscript revision. C. Adami: Conception and design of study, data collection and data analysis, manuscript revision.

## Conflict of interest

Dr Adami is an Associate Editor of the *Journal of Small Animal Practice* and co‐author of this article. She was excluded from editorial decision‐making related to the acceptance of this article for publication. No other conflicts of interest have been declared.

## Data Availability

The data that support the findings of this study are available on request from the corresponding author. The data are not publicly available due to privacy or ethical restrictions.
